# Space-fractional heat transfer analysis of hybrid nanofluid along a permeable plate considering inclined magnetic field

**DOI:** 10.1038/s41598-022-09179-9

**Published:** 2022-03-25

**Authors:** Mehdi Khazayinejad, S. S. Nourazar

**Affiliations:** Department of Mechanical Engineering, Amirkabir University of Technology, Tehran, Iran

**Keywords:** Mechanical engineering, Fluid dynamics, Graphene

## Abstract

In this study, the Caputo space-fractional derivatives of energy equation are used to model the heat transfer of hybrid nanofluid flow along a plate. The plate is considered permeable and affected by an inclined magnetic field. We use the space-fractional derivative of Fourier’s law to communicate between the nonlocal temperature gradient and heat flux. The hybrid nanofluid is formed by dispersing graphene oxide and silver nanoparticles in water. The new fractional integro-differential boundary layer equations are reduced to ordinary nonlinear equations utilizing suitable normalizations and solved via a novel semi-analytical approach, namely the optimized collocation method. The results reveal that the increment of the order of space-fractional derivatives and the magnetic inclination angle increase the Nusselt number. Also, an increase in the order of space-fractional derivatives leads to a thicker thermal boundary layer thickness resulting in a higher temperature. It is also found that the temperature of the fluid rises by changing the working fluid from pure water to single nanofluid and hybrid nanofluid, respectively. What is more, the proposed semi-analytical method will be beneficial to future research in fractional boundary layer problems.

## Introduction

Recently, energy-saving in heat transfer systems by hybrid nanofluid has attracted attention from scholars^1–4^. A hybrid nanofluid is a new type of working fluid that consists of two or more nanoparticles. The nanoparticles in a hybrid nanofluid interact synergistically and simultaneously instead of a single nanoparticle in the conventional fluid. The hybrid nanofluid has a broad range of engineering and industrial applications, such as solar thermal systems, cooling of the electronic components, biomedical applications, heat exchangers, machining, heat pipes, etc.^5–7^. At the same heat transfer rate, using hybrid nanofluids lead to a decrease in energy consumption due to having a higher cooling capacity compared to single nanofluids and pure fluids. Bahiraei et al.^8^ examined the energy efficiency of graphene-platinum/water hybrid nanofluid flow within a tube that contains single and twin twisted tapes. Their results indicated that the hybrid nanofluid heat transport rate is higher compared to pure fluid. Rabiei et al.^9^ numerically showed that shifting the working fluid from water to graphene–platinum–water hybrid nanofluid improved the microchannel heat sink efficiency by augmenting the thermal conductivity of the base liquid. Newly, Alawi et al. experimentally^10^ studied the hybrid nanofluid flow of MWCNT/TiO2/H2O within a corrugated channel and have shown that the transport of heat is augmented by 26% when nanoparticles weight concentrations is increased by 0.1%. Using hybrid nanofluid (single-walled carbon nanotubes-silver/gasoline oil), Muhammad et al.^11^ numerically investigated the flow within the boundary layer and melting heat transport along with the thickness stretch effect. Khashi'ie et al.^12^ reported the improvement of heat transfer and the delay at the boundary layer separation point over a vertical plate due to using the hybrid nanofluid.

Magnetohydrodynamic (MHD) has great potential in industrial applications such as electromagnetic casting, MHD generators, fusion reactors, biological systems, pumping, crystal growth process, MHD accelerators, etc. Newly, many experimental works have been done on the MHD systems. Bühler et al.^13^ performed an experimental study on the pressure drop of liquid metal flow in channel inserts that are exposed to a magnetic field. Zhao et al.^14^ experimentally studied a thrust vector system to check the effect of magnetic field on the deflection of plasma jet and energy extraction. Most researchers have studied the effects of magnetic fields on fluid flow under a constant angle in which the magnetic field is perpendicular to the boundary surfaces. However, the fluid flow subject to an inclined magnetic field is a more challenging task. Recently, controlling fluid flow and heat transport by applying different magnetic field angles has been received significant attention in practice and research. For instance, Atashafrooz et al.^15^ numerically investigated the nanofluid forced convection in a duct with an inclined magnetic field. The coupling of an inclined magnetic field with carbon nanotube-water nanofluid in a trapezoidal cavity is investigated numerically by Sayegh^16^. Dolgikh and Pavlinov^17^ carried out an experimental study on the magnetohydrodynamic pump with inclined partitions that are surrounded by ferromagnetic cores. Seyyedi et al.^18^ used the finite element method to simulate the natural convection and entropy analysis inside an enclosure under different magnetic field angles. Dadheech et al.^19^ conducted the analysis of entropy for slip flow of Williamson fluid over a stretching sheet by considering the inclined magnetic field and melting effect. Ali et al.^20^ studied the mixed convection heat transfer and oriented magnetic field on water-copper oxide nanofluid into a grooved channel using the finite element method. The study of the power-law fluid in a curvilinear cavity with an inclined magnetic field is numerically conducted using the finite element method by Hussain and Oztop^21^. Liao et al.^22^ numerically studied the effects of both inclined magnetic field and natural convection effect on the isotherms and streamlines in a square enclosure filled with water.

In recent years, the modeling of the Fourier’s law with spatial fractional derivative has been considered to increase the accuracy of modeling physical problems in various field researches^23,24^. The classical Fourier’s law describing the mechanism of thermal conduction includes the first-order derivative of the temperature that is not accurate enough to model the nanofluid behavior. New studies have shown that Fourier’s law with spatial-fractional derivatives can be used to increase the accuracy of nanofluid flow modeling^25,26^. In his model, the Fourier’s law heat flux relative to the nanofluid is calculated by a fractional order gradient in form of $q \propto \nabla^{\lambda } \overline{T} (\overline{X} ,\overline{Y} )$, where $\lambda$ is the non-integer order. The most widely used fractional derivatives are the Caputo^27–29^ and Riemann–Liouville (R–L)^30^ derivatives. Recently, Asjad et al. used time-fractional derivatives for convection flow of nanofluid between parallel plate^31^ and heat transfer of maxwell fluid over a vertical surface^32–34^. Since the terms of space derivatives are nonlinear in the transport equations, such as momentum and energy, the boundary conditions dominate the results more than the initial conditions. Table 1 reviews recent studies related to boundary layer problems that have discussed fractional derivatives through various aspects.

**Table 1:** Summary of fractional boundary layer problems presented in the literature.

Researchers	Fluid	Type of magnetic field	Case study	Type of derivative	Type of solution
Pan et al.^35^	Single nanofluid (Water-Cu Water-Ag Water-Al_2_O_3_ Water-TiO_2_)	Whitout magnetic field	Boundary layer flow in a porous media	Spatial fractional	Numerical (finite difference)
Tassaddiq^36^	Second-grade fluid	Inclined magnetic field	Boundary layer flow along an inclined heated plate	Time fractional	Numerical (Laplace along with Zakian’s algorithm)
Chen et al.^37^	Viscoelastic fluid	Vertical magnetic field	Boundary layer flow over a stretching sheet	Time fractional	Numerical (finite difference)
Yang et al.^38^	Maxwell fluid	Whitout magnetic field	stretching sheet with variable thickness	Time fractional	Numerical (finite difference)
Li et al.^39^	viscoelastic fluid	Whitout magnetic field	Boundary layer over a permeable surface	Spatial fractional	Numerical (finite difference)
Shen et al.^40^	Sisko nanofluid	Whitout magnetic field	Boundary layer flow over a continuously moving plate	Time fractional	Numerical (finite difference)
Liu et al.^41^	Maxwell fluid	Whitout magnetic field	Boundary layer over a moving plate	Time fractional	Numerical (finite difference)
Anwar et al.^42^	Single nanofluid (Water-SWCNTs Water-MWCNTs)	Vertical magnetic field	Boundary layer flow induced due to a stretching sheet	Time fractional	Numerical (Joint of finite-difference discretization and L1 algorithm)
Raza et al.^43^	Maxwell fluid	Inclined magnetic field	Boundary layer flow past an inclined accelerated plate	Time fractional	Numerical (Grave stehfest algorithm)

To the best of our knowledge, analyzing the space-fractional heat transport for the boundary layer of the hybrid nanofluid through a permeable surface under an external inclined magnetic field is not investigated yet. Here, the classical model of the energy equation is converted into the fractional model using the Caputo space-fractional derivative operator. Then the governing complex integro-differential equations are solved by a newly developed optimized collocation method. Finally, the influences of the different parameters on velocity and temperature fields are displayed graphically and discussed in detail.

## Physical model and governing equations

**Figure 1:**
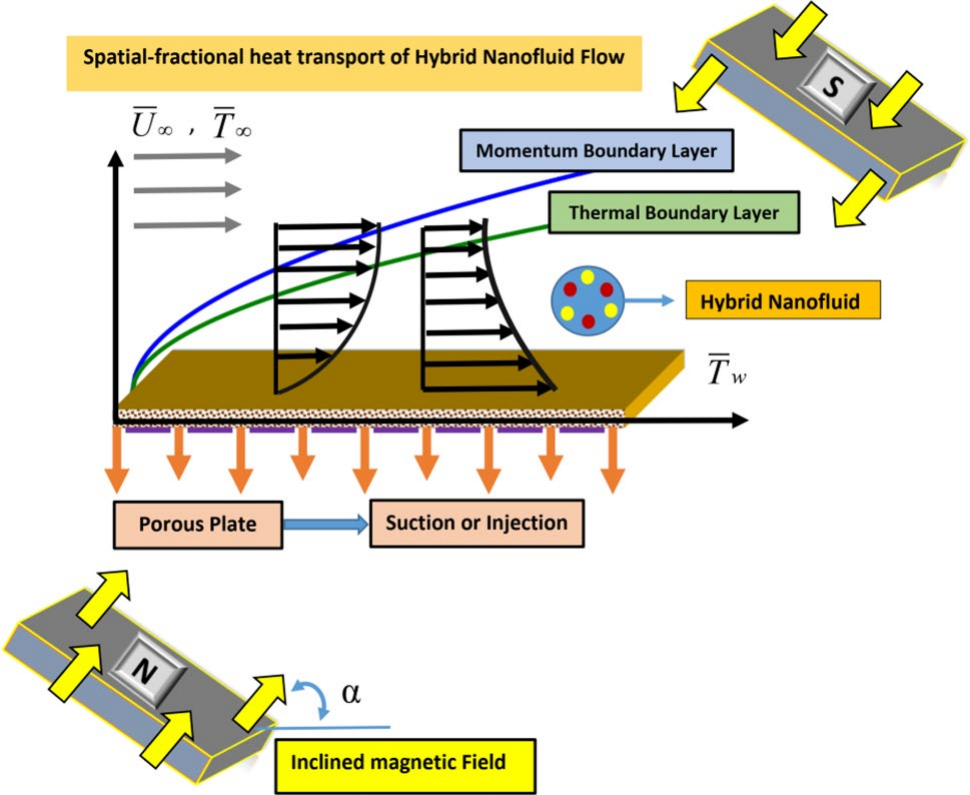
Geometry of the problem.

Let us consider the boundary layer flow of an electrically conducting hybrid nanofluid with space-fractional heat transport along a semi-infinite horizontal plate. The flow is two-dimensional, steady, incompressible, laminar, and the effects of thermal radiation and viscous dissipation are neglected. The Lorentz force is included within the momentum equation to gain Magnetohydrodynamic flow conditions. The plate is permeable, and no-slip conditions are considered. Here nanoparticles of $Ag$ $(\phi_{np1} )$ and $GO$ $(\phi_{np2} )$ are simultaneously dispersed in the $H_{2} O$ that $\phi_{np1} + \phi_{np2}$ denotes the total volume fraction of nanoparticles. We take $\overline{X}$-axis along the plate and $\overline{Y}$-axis normal to it. The plate has temperature $\overline{T}_{w}$ and injection/suction velocity through the porous plate is $\overline{V}_{w} \,(\overline{X} )$, while the temperature and velocity at external free stream are $\overline{T}_{\infty }$ and $\overline{U}_{\infty }$. It is also assumed that the flow is exposed to an external inclined magnetic field $\vec{B} = (\underbrace {B\,cos\alpha }_{{B_{{_{{\overline{X} }} }} }}\,,\,\,\underbrace {B\,sin\alpha }_{{B_{{_{{\overline{Y} }} }} }})$ with inclination angle $\alpha$ with respect to the $\overline{X}$-axis in which $\alpha$ is changed between $0^\circ$ and $90^\circ$. The physical model for this study is presented in Fig. 1.

In this study, the spatial-fractional model is proposed to modify classical Fourier’s law of thermal conduction:

1 $$ q = - k_{{\lambda_{hnf} }} \nabla^{\lambda } \overline{T} (\overline{X} ,\overline{Y} ) = - k_{{\lambda_{hnf} }} \left( {\frac{{\partial^{{^{\lambda } }} \overline{T} }}{{\partial \overline{X}^{{^{\lambda } }} }}\,\overrightarrow {i} + \frac{{\partial^{\lambda } \overline{T} }}{{\partial \overline{Y}^{{^{\lambda } }} }}\overrightarrow {j} } \right). $$

In which $q$ refers to the generalization of the classical Fourier’s law of thermal conduction, $k_{{\lambda_{hnf} }}$ signifies the generalized thermal conductivity, and $\nabla^{\lambda }$ denotes the $\lambda$ $\left( {0 < \lambda < 1} \right)$ order spatial-fractional derivative that can be defined by^44^:

2 $$ \nabla^{\lambda } \overline{T} (\overline{X} ,\overline{Y} ) = {}_{{}}^{C} D_{{\overline{X} }}^{{^{\lambda } }} \,\overline{T} (\overline{X} ,\overline{Y} )\,\,\,\overrightarrow {i} + {}_{{}}^{C} D_{{\overline{Y} }}^{{^{\lambda } }} \,\overline{T} (\overline{X} ,\overline{Y} )\,\,\overrightarrow {j} , $$

here ${}_{{}}^{C} D_{{\overline{X} }}^{{^{\alpha } }}$ and ${}_{{}}^{C} D_{{\overline{Y} }}^{{^{\alpha } }}$ stand for operators of Caputo’s spatial-fractional derivatives. Applying these operators from^44^, we have:

3 $$ \nabla^{\lambda } \overline{T} (\overline{X} ,\overline{Y} ) = \left\{ \begin{gathered} \frac{1}{\Gamma (1 - \lambda )}\,\left[ {\left( {\int\limits_{0}^{{\overline{X} }} {\frac{{\frac{{\partial \overline{T} (\zeta ,\overline{Y} )}}{\partial \zeta }}}{{(\overline{X} - \zeta )^{\lambda } }}d\zeta } } \right)\,\,\overrightarrow {i} + \left( {\int\limits_{0}^{{\overline{Y} }} {\frac{{\frac{{\partial \overline{T} (\overline{X} ,\zeta )}}{\partial \zeta }}}{{(\overline{Y} - \zeta )^{\lambda } }}d\zeta } \,} \right)\,\,\overrightarrow {j} } \right],\,\,\,\,\,\,\,\,\,0 < \lambda < 1 \hfill \\ \frac{{\partial \overline{T} }}{{\partial \overline{X} }}\,\overrightarrow {i} + \frac{{\partial \overline{T} }}{{\partial \overline{Y} }}\overrightarrow {j} ,\,\,\,\,\,\,\,\,\,\,\,\,\,\,\,\,\,\,\,\,\,\,\,\,\,\,\,\,\,\,\,\,\,\,\,\,\,\,\,\,\,\,\,\,\,\,\,\,\,\,\,\,\,\,\,\,\,\,\,\,\,\,\,\,\,\,\,\,\,\,\,\,\,\,\,\,\,\,\,\,\,\lambda = 1 \hfill \\ \end{gathered} \right., $$

Here $\Gamma (.)$ denotes the Gamma function and can be given by:

4 $$ \Gamma (x) = \int\limits_{0}^{\infty } {e^{ - p} p^{x - 1} dp} . $$

The interaction between the magnetic field and the fluid flow causes the generation of Lorentz body force:

5 $$ F_{L} = \vec{J} \times \vec{B}, $$

in which the $F_{L}$ is known as the Lorentz force and $B = B_{0} \overline{X}^{ - 0.5}$ highlights the intensity magnetic field . Also $\vec{J}$ denotes current density vector where can be given as:

6 $$ \vec{J}\, = \sigma_{hnf} \left( {\vec{V} \times \vec{B}} \right) = \sigma_{hnf} \,B\left( {\overline{U} \,\,sin\alpha - \overline{V} \,\,cos\alpha } \right)\vec{k}, $$

in which $\alpha$ is the inclination angle of the magnetic field, $\sigma_{hnf}$ represents the electrical conductivity, $\vec{V}$ signifies the velocity vector and $(\overline{U} ,\overline{V} )$ refer to the velocity components along the axes $(\overline{X} ,\overline{Y} )$, respectively. Thus, the Lorentz force can be expressed in the form^45,46^:

7 $$ F_{L} = \left( {\underbrace {{\sigma B^{2} \overline{V} \,\,cos\alpha \,\,sin\alpha - \sigma B^{2} \overline{U} \,\,sin^{2} \alpha }}_{{F_{{L_{{\overline{X} }} }} }}\,,\,\,\underbrace {{\sigma B^{2} \overline{U} \,cos\alpha \,\,sin\alpha - \sigma B^{2} \overline{V} \,\,cos^{2} \alpha }}_{{F_{{L_{{\overline{Y} }} }} }}} \right)\,. $$

Using the aforesaid assumptions, the conservation equations for mass, momentum, and spatial-fractional derivatives of energy may be formulated by^23,25,47^:

8 $$ \frac{{\partial \overline{U} }}{{\partial \overline{X} }} + \frac{{\partial \overline{V} }}{{\partial \overline{Y} }} = 0, $$

9 $$ \rho_{hnf} \left( {\overline{U} \frac{{\partial \overline{U} }}{{\partial \overline{X} }} + \overline{V} \frac{{\partial \overline{U} }}{{\partial \overline{Y} }}} \right) = \mu_{hnf} \left( {\frac{{\partial^{{^{2} }} \overline{U} }}{{\partial \overline{X}^{{^{2} }} }} + \frac{{\partial^{{^{2} }} \overline{U} }}{{\partial \overline{Y}^{{^{2} }} }}} \right)\, + \sigma_{hnf} B^{2} \overline{V} \,\,cos\alpha \,\,sin\alpha + \sigma_{hnf} B^{2} \left( {\overline{U}_{\infty } - \,\,\overline{U} } \right)\,\,sin^{2} \alpha , $$

10 $$ \rho_{hnf} \left( {\overline{U} \frac{{\partial \overline{V} }}{{\partial \overline{X} }} + \overline{V} \frac{{\partial \overline{V} }}{{\partial \overline{Y} }}} \right) = \mu_{hnf} \left( {\frac{{\partial^{{^{2} }} \overline{V} }}{{\partial \overline{X}^{{^{2} }} }} + \frac{{\partial^{{^{2} }} \overline{V} }}{{\partial \overline{Y}^{{^{2} }} }}} \right) + \sigma B^{2} \overline{U} \,cos\alpha \,\,sin\alpha - \sigma B^{2} \overline{V} \,\,cos^{2} \alpha , $$

11 $$ \left( {\rho c_{p} } \right)_{hnf} \left( {\overline{U} \frac{{\partial \overline{T} }}{{\partial \overline{X} }} + \overline{V} \frac{{\partial \overline{T} }}{{\partial \overline{Y} }}} \right) = \frac{{k_{{\lambda_{hnf} }} }}{\Gamma (1 - \lambda )}\,\left[ {\,\,\left( {\int\limits_{0}^{{\overline{X} }} {\frac{{\frac{{\partial^{2} \overline{T} (\zeta ,\overline{Y} )}}{{\partial \zeta^{2} }}}}{{(\overline{X} - \zeta )^{\lambda } }}} d\zeta } \right)\, + \left( {\int\limits_{0}^{{\overline{Y} }} {\frac{{\frac{{\partial^{2} \overline{T} (\overline{X} ,\zeta )}}{{\partial \zeta^{2} }}}}{{(\overline{Y} - \zeta )^{\lambda } }}} d\zeta \,} \right)\,\,} \right]. $$

with subjected boundary conditions as^48–50^:

12 $$ \overline{U} = 0,\,\,\,\,\,\,\,\,\overline{V} = \overline{V}_{w} \,(\overline{X} ),\,\,\,\,\,\,\,\,\overline{T} = \overline{T}_{w} \,\,\,\,\,\,\,\,\,\,\,\,at\,\,\,\,\,\,\,\,\overline{Y} = 0, $$

13 $$ \overline{U} \to \overline{U}_{\infty } ,\,\,\,\,\,\,\,\,\overline{T} \to \overline{T}_{\infty } \,\,\,\,\,\,\,\,\,\,\,\,as\,\,\,\,\,\,\,\,\overline{Y} \to \infty , $$

In the above equations $\overline{P}$ is the pressure, $\lambda$ refers to the fractional order and $\overline{T}$ indicates the temperature. Moreover, the density $\rho_{hnf}$, heat capacity $c_{{p_{hnf} }}$, viscosity $\mu_{hnf}$, thermal conductivity $k_{{\lambda_{hnf} }}$, and electrical conductivity $\sigma_{hnf}$ of the hybrid nanofluid are obtained using the following expressions^51,52^:

14 $$ \rho_{hnf} = \left( {1 - \phi_{np2} } \right)\left( {\left( {1 - \phi_{np1} } \right)\rho_{f} + \phi_{np1} \rho_{np1} } \right) + \phi_{np2} \rho_{np2} , $$

15 $$ \left( {\rho c_{p} } \right)_{hnf} = \left( {1 - \phi_{np2} } \right)\left( {\left( {1 - \phi_{np1} } \right)\left( {\rho c_{p} } \right)_{f} + \phi_{np1} \left( {\rho c_{p} } \right)_{np1} } \right) + \phi_{np2} \left( {\rho c_{p} } \right)_{np2} , $$

16 $$ \mu_{hnf} = \frac{{\mu_{f} }}{{\left( {1 - \phi_{np1} } \right)^{2.5} \left( {1 - \phi_{np2} } \right)^{2.5} }}, $$

17 $$ \frac{{k_{{\lambda_{hnf} }} }}{{k_{nf} }} = \omega \frac{{k_{np2} + 2{\mkern 1mu} k_{nf} - 2\phi_{np2} \left( {k_{nf} - k_{np2} } \right)}}{{k_{np2} + 2{\mkern 1mu} k_{nf} + \phi_{np2} \left( {k_{nf} - k_{np2} } \right)}}, $$

18 $$ \frac{{\sigma_{hnf} }}{{\sigma_{nf} }} = \frac{{\sigma_{np2} \left( {1 + 2{\mkern 1mu} \phi_{np2} } \right) + 2\sigma_{nf} \left( {1 - \phi_{np2} } \right)}}{{\sigma_{np2} \left( {1 - \phi_{np2} } \right) + \sigma_{nf} \left( {2 + \phi_{np2} } \right)}}. $$

To calculate the thermal conductivity ($k_{{\lambda_{hnf} }}$) and the electrical conductivity ($\sigma_{hnf}$) of the hybrid nanofluid, the values of $k_{nf}$ and $\sigma_{nf}$ must be replaced by $k_{f} \frac{{k_{np1} + 2{\mkern 1mu} k_{f} - 2{\mkern 1mu} \phi_{np1} \left( {k_{f} - k_{np1} } \right)}}{{k_{np1} + 2{\mkern 1mu} k_{f} + \phi_{np1} \left( {k_{f} - k_{np1} } \right)}}$ and $\sigma_{f} \frac{{\sigma_{np1} \left( {1 + 2{\mkern 1mu} \phi_{np1} } \right) + 2\sigma_{f} \left( {1 - \phi_{np1} } \right)}}{{\sigma_{np1} \left( {1 - \phi_{np1} } \right) + \sigma_{f} \left( {2 + \phi_{np1} } \right)}}$ in Eqs. (17) and (18), respectively. The coefficient $\omega$ in Eq. (17) balances the dimension of Eq. (1), which $\omega = 1$ is considered here. Table 2 gives the thermo-physical properties of nanoparticles $GO$ and $Ag$ and fluid phase $\left( {H_{2} O} \right)$.

**Table 2:** Thermo-physical properties of nanoparticles and fluid phase^53,54^.

Properties	$c_{p} \,\left( {{J \mathord{\left/ {\vphantom {J {kg\,K}}} \right. \kern-\nulldelimiterspace} {kg\,K}}} \right)$	$\rho \,\left( {{{kg} \mathord{\left/ {\vphantom {{kg} {m^{3} }}} \right. \kern-\nulldelimiterspace} {m^{3} }}} \right)$	$\mu \,({{kg} \mathord{\left/ {\vphantom {{kg} {m\,s}}} \right. \kern-\nulldelimiterspace} {m\,s}}\,)$	$\sigma \,\left( {{1 \mathord{\left/ {\vphantom {1 {\Omega m}}} \right. \kern-\nulldelimiterspace} {\Omega m}}} \right)$	$k\,\left( {{W \mathord{\left/ {\vphantom {W {m\,K}}} \right. \kern-\nulldelimiterspace} {m\,K}}} \right)$
Water	4179	997.1	1.003 × 10^–3^	0.05	0.613
Graphene oxide	717	1800	–	1.1 × 10^–5^	5000
Silver	235	10,500	–	6.30 × 10^7^	429

The governing equations may be simplified by introducing the following similarity transformation variables^48,50^:

19 $$ \eta = \overline{Y} \sqrt {\frac{{\overline{U}_{\infty } }}{{\nu_{f} \overline{X} }}} ,\,\,\,\,\,\,\,\,f = \frac{{ - \psi (\overline{X} ,\overline{Y} )}}{{\sqrt {\nu_{f} \overline{X} \,\overline{U}_{\infty } } }},\,\,\,\,\,\,\,\,\theta = \frac{{\overline{T} - \overline{T}_{\infty } }}{{\overline{T}_{w} - \overline{T}_{\infty } }}, $$

where $f$ and $\theta$ are the dimensionless stream function and temperature. Also, $\psi$ indicates the stream function which can be determined as:

20 $$ \left( {\overline{U} ,\overline{V} } \right) = \left( {\frac{ - \partial \psi }{{\partial \overline{Y} }},\frac{\partial \psi }{{\partial \overline{X} }}} \right). $$

The continuity equation Equation (Eq. (8)) is automatically satisfied by defining the stream function. On the other hand, based on Eqs. (15) and (16) we can infer:

21 $$ {\raise0.7ex\hbox{${\overline{U} }$} \!\mathord{\left/ {\vphantom {{\overline{U} } {\overline{U}_{\infty } }}}\right.\kern-\nulldelimiterspace} \!\lower0.7ex\hbox{${\overline{U}_{\infty } }$}} = f^{\prime}\left( \eta \right),\,\,\,\,\,\,\,\,\overline{V} = \sqrt {\frac{{\nu_{f} \overline{U}_{\infty } }}{{4\overline{X} }}} \left( {\eta f^{\prime}\left( \eta \right) - f\left( \eta \right)} \right), $$

where prime indicates derivation with respect to $\eta$. Applying the above transformations leads to gaining the injection/suction velocity:

22 $$ \overline{V}_{w} \,(\overline{X} ) = \frac{ - f\left( 0 \right)}{2}\sqrt {\frac{{\nu_{f} \overline{U}_{\infty } }}{{\overline{X} }}} . $$

Using the mentioned similarity transformations, Caputo’s spatial-fractional derivative model, and boundary layer approximations, Eqs. (9) and (11) may be written in forms as:

23 $$ f^{\prime\prime\prime}\left( \eta \right) + \frac{1}{2}\frac{{\rho_{hnf} }}{{\rho_{f} }}\frac{{\mu_{f} }}{{\mu_{hnf} }}f\left( \eta \right)f^{\prime\prime}\left( \eta \right) + Ha\,\,sin^{2} \alpha \,\,\frac{{\sigma_{hnf} }}{{\sigma_{f} }}\frac{{\mu_{f} }}{{\mu_{hnf} }}\left( {1 - f^{\prime}\left( \eta \right)} \right) = 0, $$

24 $$ \frac{{k_{{\lambda_{hnf} }} }}{{k_{f} }}\left( {\frac{{\nu_{f} \overline{X} }}{{\overline{U}_{\infty } }}} \right)^{{\frac{1 - \lambda }{2}}} \frac{1}{\Gamma (1 - \lambda )}\left( {\int\limits_{0}^{\eta } {\frac{{\frac{{d^{2} \theta (\tau )}}{{d\tau^{2} }}}}{{(\eta - \tau )^{\lambda } }}d\tau } \,} \right)\,\, + \frac{Pr}{2}\frac{{\left( {\rho c_{p} } \right)_{hnf} }}{{\left( {\rho c_{p} } \right)_{f} }}f\left( \eta \right)\theta^{\prime}\left( \eta \right) = 0. $$

The converted boundary conditions for Eqs. (23) and (24) take the form^50^:

25 $$ \begin{gathered} f\left( 0 \right) = S,\,\,\,\,\,\,\,\,f^{\prime}\left( 0 \right) = 0,\,\,\,\,\,\,\,\,\theta \left( 0 \right) = 1, \hfill \\ \hfill \\ f^{\prime}\, \to 1,\,\,\,\,\,\,\,\,\theta \to 0\,\,\,\,\,\,\,\,\,\,\,\,as\,\,\,\,\,\,\,\,\eta \to \infty , \hfill \\ \end{gathered} $$

where the $Ha = \frac{{\sigma_{f} B_{0}^{2} }}{{\rho_{f} \overline{U}_{\infty } }}$ indicates the magnetic parameter (Hartmann number), $Pr = \frac{{\nu_{f} \left( {\rho \,c_{p} } \right)_{f} }}{{k_{f} }}$ shows the Prandtl number and $S = - 2\frac{{\overline{V}_{w} (\overline{X} )}}{{\overline{U}_{\infty } }}\,{\text{Re}}^{0.5}$ signifies the injection/suction parameter which $S > 0$ and $S < 0$ are for the mass suction and mass injection, respectively.

Two physical parameters that play an important role in engineering processes are the shear stress coefficient $C_{f}$ and the Nusselt number $Nu$ that are obtained as:

26 $$ C_{f} = \,\frac{{\tau_{w} }}{{\frac{1}{2}\rho_{f} \overline{U}_{\infty }^{2} }},\,\,\,\,\,\,\,\,Nu = \,\frac{{\overline{X} q_{w} }}{{k_{f} \left( {\overline{T}_{w} - \overline{T}_{\infty } } \right)}}, $$

in which $\tau_{w}$ and $q_{w}$ respectively represent the surface shear stress and heat flux that can be expressed as:

27 $$ \tau_{w} = \,\mu_{hnf} \left( {\frac{{\partial \overline{U} }}{{\partial \overline{Y} }}} \right)_{{\overline{Y} = 0}} ,\,\,\,\,\,\,\,\,q_{w} = \, - k_{{\lambda_{hnf} }} \left( {\frac{{\partial^{{^{\lambda } }} \overline{T} }}{{\partial \overline{Y}^{{^{\lambda } }} }}} \right)_{{\overline{Y} = 0}} , $$

Utilizing Eq. (27) into Eq. (26) and upon substitution of non-dimensional variables, we get:

28 $$ C_{{f_{r} }} = C_{f} \sqrt {Re} = \frac{2}{{\left( {1 - \phi_{np1} } \right)^{2.5} \left( {1 - \phi_{np2} } \right)^{2.5} }}f^{\prime\prime}\left( 0 \right)\,, $$

29 $$ Nu_{r} = \frac{Nu}{{\sqrt {\text{Re}} }} = \, - \frac{{k_{{\lambda_{hnf} }} }}{{k_{f} }}\frac{1}{\Gamma (1 - \lambda )}\left( {\frac{{\upsilon_{f} \overline{X} }}{{U_{\infty } }}} \right)^{{\frac{1 - \lambda }{2}}} \left( {\int\limits_{0}^{\eta } {\frac{{\frac{d\theta (\zeta )}{{d\zeta }}}}{{(\eta - \zeta )^{\lambda } }}d\zeta } \,} \right)_{{_{\eta = 0} }} , $$

where $Re = \frac{{\overline{U}_{\infty } \overline{X} }}{{\nu_{f} }}$ signifies the local Reynolds number.

## Solution methodology

In the following section, an efficient semi-analytical scheme, optimal collocation method (OCM) is used to investigate the problem defined by Eqs. (23) and (27). Khazayinejad et al.^47,55^ and Nourazar et al.^56^ proposed this method to optimize the collocation method (CM)^57,58^ and applied it to nonlinear problems involving infinite boundary conditions. The proposed method involves seven steps. In the first step, the interval $0 \le \eta < \infty$ is converted to $0 \le \eta \le \eta_{\infty }$, which $\eta_{\infty }$ changes with different physical parameters. In the second step, the physical domain is normalized to a computational domain by:

30 $$ 0 \le \eta \le \eta_{\infty } \mathop \Rightarrow \limits^{{u = {\eta \mathord{\left/ {\vphantom {\eta {\eta_{\infty } }}} \right. \kern-\nulldelimiterspace} {\eta_{\infty } }}}} 0 \le u \le 1. $$

In the third step, using normalizations $G\left( u \right) = {{f\left( \eta \right)} \mathord{\left/ {\vphantom {{f\left( \eta \right)} {\eta_{\infty } }}} \right. \kern-\nulldelimiterspace} {\eta_{\infty } }}$ and $Q\left( u \right) = {{\theta \left( \eta \right)} \mathord{\left/ {\vphantom {{\theta \left( \eta \right)} {\eta_{\infty } }}} \right. \kern-\nulldelimiterspace} {\eta_{\infty } }}$, the Eqs. (23) and (24) are rewritten as:

31 $$ \frac{1}{{\eta_{\infty }^{2} }}G^{\prime\prime\prime}\left( u \right) + \frac{1}{2}\frac{{\rho_{hnf} }}{{\rho_{f} }}\frac{{\mu_{f} }}{{\mu_{hnf} }}G\left( u \right)G^{\prime\prime}\left( u \right) + Ha\,sin^{2} \alpha \,\frac{{\sigma_{hnf} }}{{\sigma_{f} }}\frac{{\mu_{f} }}{{\mu_{hnf} }}\left( {1 - G^{\prime}\left( u \right)} \right) = 0, $$

32 $$ \frac{1}{{\eta_{\infty }^{\lambda } }}\frac{{k_{{\lambda_{hnf} }} }}{{k_{f} }}\left( {\frac{{\nu_{f} \overline{X} }}{{\overline{U}_{\infty } }}} \right)^{{\frac{1 - \lambda }{2}}} \frac{1}{\Gamma (1 - \lambda )}\left( {\int\limits_{0}^{u} {\frac{{\frac{{d^{2} Q(\zeta )}}{{d\zeta^{2} }}}}{{(u - \zeta )^{\lambda } }}d\zeta } \,} \right)\,\, + \eta_{\infty } \frac{Pr}{2}\frac{{\left( {\rho c_{p} } \right)_{hnf} }}{{\left( {\rho c_{p} } \right)_{f} }}G\left( u \right)Q^{\prime}\left( u \right) = 0, $$

where primes show differentiation with respect to the $u$ ∈ [0,1]. After applying normalizations $G\left( u \right) = {{f\left( \eta \right)} \mathord{\left/ {\vphantom {{f\left( \eta \right)} {\eta_{\infty } }}} \right. \kern-\nulldelimiterspace} {\eta_{\infty } }}$ and $Q\left( u \right) = {{\theta \left( \eta \right)} \mathord{\left/ {\vphantom {{\theta \left( \eta \right)} {\eta_{\infty } }}} \right. \kern-\nulldelimiterspace} {\eta_{\infty } }}$, the corresponding new boundary conditions can be obtained as:

33 $$ \begin{gathered} G\left( 0 \right) = \frac{S}{{\eta_{\infty } }},\,\,\,\,\,\,\,\,\,\,\,G^{\prime}\left( 0 \right) = 0,\,\,\,\,\,\,\,\,G^{\prime}\left( 1 \right) = 1,\,\,\,\,\,\,\,\,\,\,\,\,\,G^{\prime\prime}\left( 1 \right) = 0, \hfill \\ Q\left( 0 \right) = \frac{1}{{\eta_{\infty } }},\,\,\,\,\,\,\,\,Q\left( 1 \right) = 0,\,\,\,\,\,\,\,\,\,\,Q^{\prime}\left( 1 \right) = 0. \hfill \\ \end{gathered} $$

The idea for choice $Q^{\prime}\left( 1 \right) = 0$ and $G^{\prime\prime}\left( 1 \right) = 0$ comes from the asymptotic conditions. In the fourth step, to determine a solution for the Eqs. (33) and (34), two trial solutions with unfamiliar coefficients “c” will be selected that have the following form^45,59^:

34 $$ G(u) = \frac{1}{{\eta_{\infty } }}\left( {{\text{c}}_{0} + \sum\limits_{j = 1}^{k} {c_{j} } \,u^{j} } \right) = \frac{1}{{\eta_{\infty } }}\left( {c_{0} + c_{1} \,u + c_{2} \,u^{2} + \ldots + c_{k} \,u^{k} } \right), $$

35 $$ Q(u) = \frac{1}{{\eta_{\infty } }}\left( {{\text{c}}_{k + 1} + \sum\limits_{j = 1}^{m} {c_{j + k + 1} } \,u^{\lambda j} } \right) = \frac{1}{{\eta_{\infty } }}\left( {c_{k + 1} + c_{k + 2} \,u^{\lambda } + c_{k + 3} \,u^{2\lambda } + \ldots + c_{m + k + 1} \,u^{m\,\lambda } } \right). $$

The accuracy of the trial solutions increases with the consideration of more terms in the above series. Note that the OCM, unlike the CM, gives us a lot of freedom and flexibility to choose trial solutions. Because in this method, there is no need for unfamiliar unknowns and weight functions to be equal in number. The weight functions corresponding to trial solutions are obtained from the following relation:

36 $$ n_{{W_{j} }} = n_{{c_{j} }} + 1 - n_{a} - n_{bc} . $$

In above equation $n_{a}$, $n_{bc}$, $n_{{c_{j} }}$, $n_{{W_{j} }}$ signify the number of asymptotic boundary conditions, boundary conditions, unfamiliar constants, and weight functions. Using Eq. (35), the new relations can be obtained as:

37 $$ u = 0\,\,\,\,\, \Rightarrow \,\,\,\,\,G = \frac{S}{{\eta_{\infty } }}\,\,\,\,\,\,\,\,\, \Rightarrow \,\,\,\,\,\,\,\,\,\,\,\,\,c_{0} = S, $$

38 $$ u = 0\,\,\,\,\, \Rightarrow \,\,\,\,\,G^{\prime} = 0\,\,\,\,\,\,\,\,\,\,\, \Rightarrow \,\,\,\,\,\,\,\,\,\,\,\,\,c_{1} = 0, $$

39 $$ u = 1\,\,\,\,\, \Rightarrow \,\,\,\,\,G^{\prime} = 1\,\,\,\,\,\,\,\,\,\,\,\,\, \Rightarrow \,\,\,\,\,\,\,\,\,\,\,\,\,\frac{1}{{\eta_{\infty } }}\left( {c_{1} \, + 2c_{2} + \ldots + kc_{k} } \right) = 1, $$

40 $$ u = 0\,\,\,\,\, \Rightarrow \,\,\,\,\,Q = \frac{1}{{\eta_{\infty } }}\,\,\,\,\,\,\,\,\, \Rightarrow \,\,\,\,\,\,\,\,\,\,\,\,\,c_{k + 1} = 1, $$

41 $$ u = 1\,\,\,\,\, \Rightarrow \,\,\,\,\,Q = 0\,\,\,\,\,\,\,\,\,\,\,\,\,\, \Rightarrow \,\,\,\,\,\,\,\,\,\,\,\,\,c_{k + 1} + c_{k + 2} \, + c_{k + 3} \, + \ldots + c_{m + k + 1} = 0, $$

42 $$ u = 1\,\,\,\,\, \Rightarrow \,\,\,\,\,G^{\prime\prime} = 0\,\,\,\,\,\,\,\,\,\,\, \Rightarrow \,\,\,\,\,\,\,\,\,\,\,\,\,2c_{2} + 6c_{3} + \ldots + k(k - 1)c_{k} = 0, $$

43 $$ u = 1\,\,\,\,\, \Rightarrow \,\,\,\,\,Q^{\prime} = 0\,\,\,\,\,\,\,\,\,\,\,\,\, \Rightarrow \,\,\,\,\,\,\,\,\,\,\,\,\,c_{k + 2} + 2c_{k + 3} + \ldots + mc_{m + k + 1} = 0. $$

In the fifth step, the residual functions $R_{G} \left( {c_{0} ,c_{1} ,...,c_{k} } \right)$ and $R_{Q} \left( {c_{0} ,c_{1} ,...,c_{m + k + 1} } \right)$ must be calculated. These functions can be obtained by placing $g$ and $h$ into Eqs. (33) and (34):

44 $$ \begin{gathered} R_{G} \left( {c_{0} ,c_{1} ,...,c_{k} } \right) = \frac{1}{{\eta_{\infty }^{3} }}\frac{{d^{3} }}{{du^{3} }}\left( {{\text{c}}_{0} + \sum\limits_{j = 1}^{k} {c_{j} } \,u^{j} } \right) + \frac{1}{{2\eta_{\infty }^{2} }}\frac{{\rho_{hnf} }}{{\rho_{f} }}\frac{{\mu_{f} }}{{\mu_{hnf} }}\left( {{\text{c}}_{0} + \sum\limits_{j = 1}^{k} {c_{j} } \,u^{j} } \right) \hfill \\ \frac{{d^{2} }}{{du^{2} }}\left( {{\text{c}}_{0} + \sum\limits_{j = 1}^{k} {c_{j} } \,u^{j} } \right) + Ha\,sin^{2} \alpha \frac{{\sigma_{hnf} }}{{\sigma_{f} }}\frac{{\mu_{f} }}{{\mu_{hnf} }}\left( {1 - \frac{1}{{\eta_{\infty } }}\frac{d}{du}\left( {{\text{c}}_{0} + \sum\limits_{j = 1}^{k} {c_{j} } \,u^{j} } \right)} \right), \hfill \\ \end{gathered} $$

45 $$ \begin{gathered} R_{Q} \left( {c_{0} ,c_{1} ,...,c_{m + k + 1} } \right) = \frac{{k_{{\lambda_{hnf} }} }}{{\eta_{\infty }^{\lambda + 1} \,k_{f} \,\Gamma (1 - \lambda )}}\left( {\frac{{\nu_{f} \overline{X} }}{{\overline{U}_{\infty } }}} \right)^{{\frac{1 - \lambda }{2}}} \int\limits_{0}^{u} {\frac{{\frac{{d^{2} }}{{d\zeta^{2} }}\left( {{\text{c}}_{k + 1} + \sum\limits_{j = 1}^{m} {c_{j + k + 1} } \,\zeta^{\lambda j} } \right)}}{{(u - \zeta )^{\lambda } }}d\zeta } \hfill \\ + \frac{1}{{\eta_{\infty } }}\frac{Pr}{2}\frac{{\left( {\rho c_{p} } \right)_{hnf} }}{{\left( {\rho c_{p} } \right)_{f} }}\left( {{\text{c}}_{0} + \sum\limits_{j = 1}^{k} {c_{j} } \,u^{j} } \right)\frac{d}{du}\left( {{\text{c}}_{k + 1} + \sum\limits_{j = 1}^{m} {c_{j + k + 1} } \,u^{\lambda j} } \right).\,\,\,\,\,\,\,\,\,\,\,\,\,\,\,\,\,\,\,\,\,\,\,\,\,\,\,\,\,\,\,\,\,\,\,\,\,\,\,\,\,\,\,\,\,\,\,\,\,\,\,\,\,\,\,\,\,\,\,\, \hfill \\ \end{gathered} $$

Further, in this method, the sum of weighted residual values must be zero in the problem domain:

46 $$ \int_{u} {\,R(u)\,\,\,W_{j} (u)} \, = 0\,\,\,\,\,,j = 1,2,...,k\,, $$

which $W_{i} (u)$ is the weight function and can be selected by:

47 $$ W_{j} (u) = \delta (u - u_{j} ), $$

which $\delta (u - u_{i} )$ represents the Dirac delta function. In the sixth step, collocation points are selected that leads to the following results:

48 $$ R_{G} \left( {\frac{1}{k - 1}} \right) = 0\,\,\,\,\,\,\,\,\,\,\,\,,\,\,\,\,\,\,\,\,\,\,\,R_{G} \left( {\frac{2}{k - 1}} \right) = 0\,\,\,\,\,\,\,\,\,\,\,\,,\,\,\,\,\,\,\,\,\,\,\,R_{G} \left( {\frac{3}{k - 1}} \right) = 0\,\,\,\,\,\,\,\,\,\,\,\,,...,\,\,\,\,\,\,\,\,\,\,\,R_{G} \left( {\frac{k - 2}{{k - 1}}} \right) = 0, $$

49 $$ R_{Q} \left( {\frac{1}{m - 1}} \right) = 0\,\,\,\,\,\,\,\,\,\,\,\,,\,\,\,\,\,\,\,\,\,\,\,R_{Q} \left( {\frac{2}{m - 1}} \right) = 0\,\,\,\,\,\,\,\,\,\,\,\,,\,\,\,\,\,\,\,\,\,\,\,R_{Q} \left( {\frac{3}{m - 1}} \right) = 0\,\,\,\,\,\,\,\,\,\,\,\,,...,\,\,\,\,\,\,\,\,\,\,\,R_{Q} \left( {\frac{m - 2}{{m - 1}}} \right) = 0. $$

In the seventh step, the obtained equations from previous steps are solved. Here, Eqs. (39)–(43) and Eqs. (48)–(49) leads to a system of $k + m + 3$ algebraic equations. Thus, By solving the relevant equations, we can easily find the unknown coefficients $c_{j}$ and $\eta_{\infty }$. By using these values in Eqs. (34) and (35), the velocity and temperature distributions can be computed. for a special case, the results of the approximate solutions for $GO - Ag - H_{2} O$ hybrid nanofluid when $Ha = 1$, $\lambda = 0.94$, $\alpha = \frac{\pi }{4}$, $\overline{X} = 1$, $\phi_{np1} = 0.03$, $\phi_{np2} = 0.03$, $S = 0.3$, $k = 12$, and $m = 12$ are presented as:

52 $$ f\left( \eta \right) = 0.3 + 0.4412537794\,{\mkern 1mu} \eta^{2} - 0.1081092858{\mkern 1mu} \,\eta^{3} + \ldots - 0.0000000038{\mkern 1mu} \,\eta^{12} , $$

51 $$ \theta \left( \eta \right) = 1 - 1.733723899{\mkern 1mu} {\mkern 1mu} \,\eta^{{\frac{47}{{50}}}} + 1.048652335\,{\mkern 1mu} \eta^{{\frac{47}{{25}}}} + \ldots + 0.0000052528\,\eta^{{\frac{282}{{25}}}} . $$

## Code validation

In the following subsections, to ensure the present study's authenticity, validation with existing literature has been done in two parts:

### Validation with the previous theoretical works

The first validation is obtained by comparing the Nusselt number and skin friction coefficient with the previous theoretical works for several values of the suction parameter. The comparison is displayed in Table 3 which demonstrates an excellent agreement in all cases.

**Table 3:** Comparison results of $\left| {f^{\prime\prime}(0)} \right|$ and $\left| {\theta^{\prime}(0)} \right|$ with previous studies when $\phi_{hnp} = 0$, $Ha = {0}$, and $Pr = 0.7$.

$\frac{{\overline{V}_{w} }}{{\overline{U}_{\infty } }}\,{\text{Re}}^{0.5}$	$\left| {f^{\prime\prime}(0)} \right|$				$\left| {\theta^{\prime}(0)} \right|$				
	Bejan^60^	Schetz^61^	Schlichting^62^	Present study	Bejan^60^	Schetz^61^		Oosthuizen^63^	Present study
0	0.332	0.332	0.332	0.3321	0.292	0.295	0.293		0.2929
− 0.25	0.523	–	–	0.5228	0.429	0.429	–		0.4294
− 0.75	0.945	–	–	0.9454	0.722	0.722	–		0.7218

### Validation with the maple package

**Figure 2:**
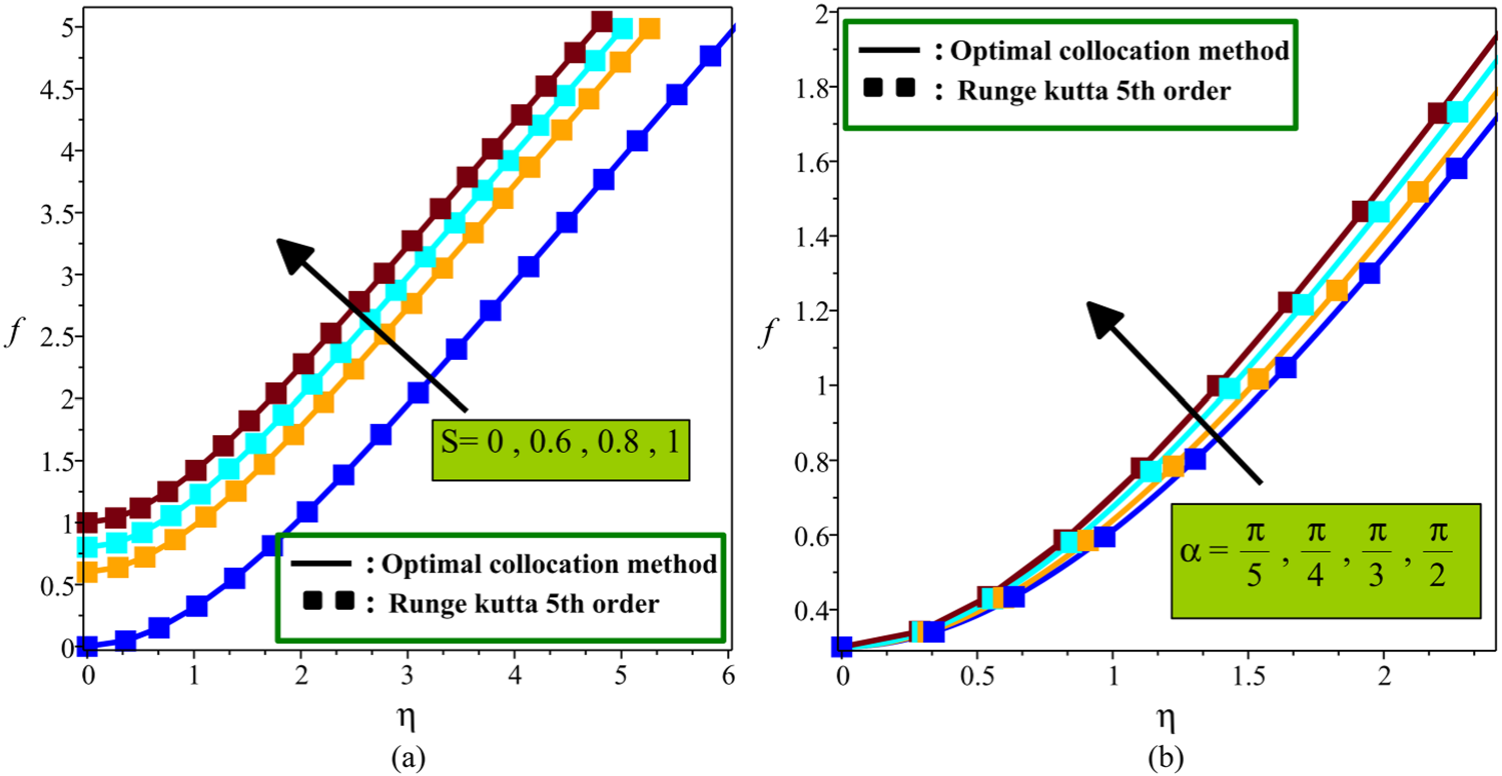
Comparison of current analysis with the numerical method at different (**a**) $S$ (**b**) $\alpha$.

The second validation is done with the Maple package through Fig. 2a,b. Maple is one of the most powerful software packages for solving nonlinear differential equations. Maple package uses an assistant with a multiple-step process for solving differential equations using the Runge–Kutta numerical method to boundary value problems. Runge–Kutta method is one of the best solving algorithms in terms of accuracy and speed of solution. So, to justify the validity of the current study, the present results (obtained from OCM) for dimensionless stream function are compared with the Runge–Kutta method for special case $\phi_{np1} = \phi_{np2} = 0.03$, $Pr = 6.84$, $Ha = 1$ and various values suction parameter $S$ (Fig. 2a) and inclination angle of the magnetic field $\alpha$ (Fig. 2b). A good agreement is seen between the results for both figures.

A comparative study of absolute error between the present method and Runge–Kutta method for $f(\eta )$ and $\theta (\eta )$ is provided in Table 4. An excellent agreement is observed from the comparison of results.

**Table 4:** Comparison between the optimal collocation method and Runge–Kutta method when $$\alpha = {\raise0.7ex\hbox{$\pi $} \!\mathord{\left/ {\vphantom {\pi 4}}\right.\kern-\nulldelimiterspace} \!\lower0.7ex\hbox{$4$}}$$$$\phi_{np1} = \phi_{np2} = 0.03$$, $$Pr = 6.84$$, $$Ha = 1$$.

$\eta$	$f(\eta )$			$\theta (\eta )$		
	Numerical	OCM	Error	Numerical	OCM	Error
0	0.30000	0.30000	0.00000	1.00000	1.00000	0.00000
0.2	0.31613	0.31611	0.00002	0.75993	0.75797	0.00196
0.4	0.36171	0.36166	0.00005	0.55866	0.55729	0.00137
0.6	0.43293	0.43285	0.00008	0.39405	0.39313	0.00092
0.8	0.52641	0.52631	0.00010	0.26457	0.26398	0.00059
1	0.63915	0.63902	0.00012	0.16780	0.16745	0.00034
2	1.40492	1.40474	0.00018	0.00580	0.00584	0.00007
3	2.34426	2.34406	0.00020	0.00002	0.00005	0.00003
4	3.33370	3.33350	0.00020	0.00000	0.00000	0.00000
5.0	4.33254	4.33234	0.00019			
5.79	5.12850	5.12831	0.00019			

## Results and discussions

In this research, to offer the influences of different parameters on flow and heat transport characteristics, results have been shown in terms of streamline contours, skin friction coefficient, nonlocal Nusselt number, and velocity and temperature distributions. Throughout the study, the parameters default values are taken as $\phi_{np1} = 0.03$, $\phi_{np2} = 0.03$ $Pr = 6.84$, $\alpha = {\pi \mathord{\left/ {\vphantom {\pi 4}} \right. \kern-\nulldelimiterspace} 4}$, $Ha = 1$, $S = 0.3$, and $\lambda = 0.94$.

**Figure 3:**
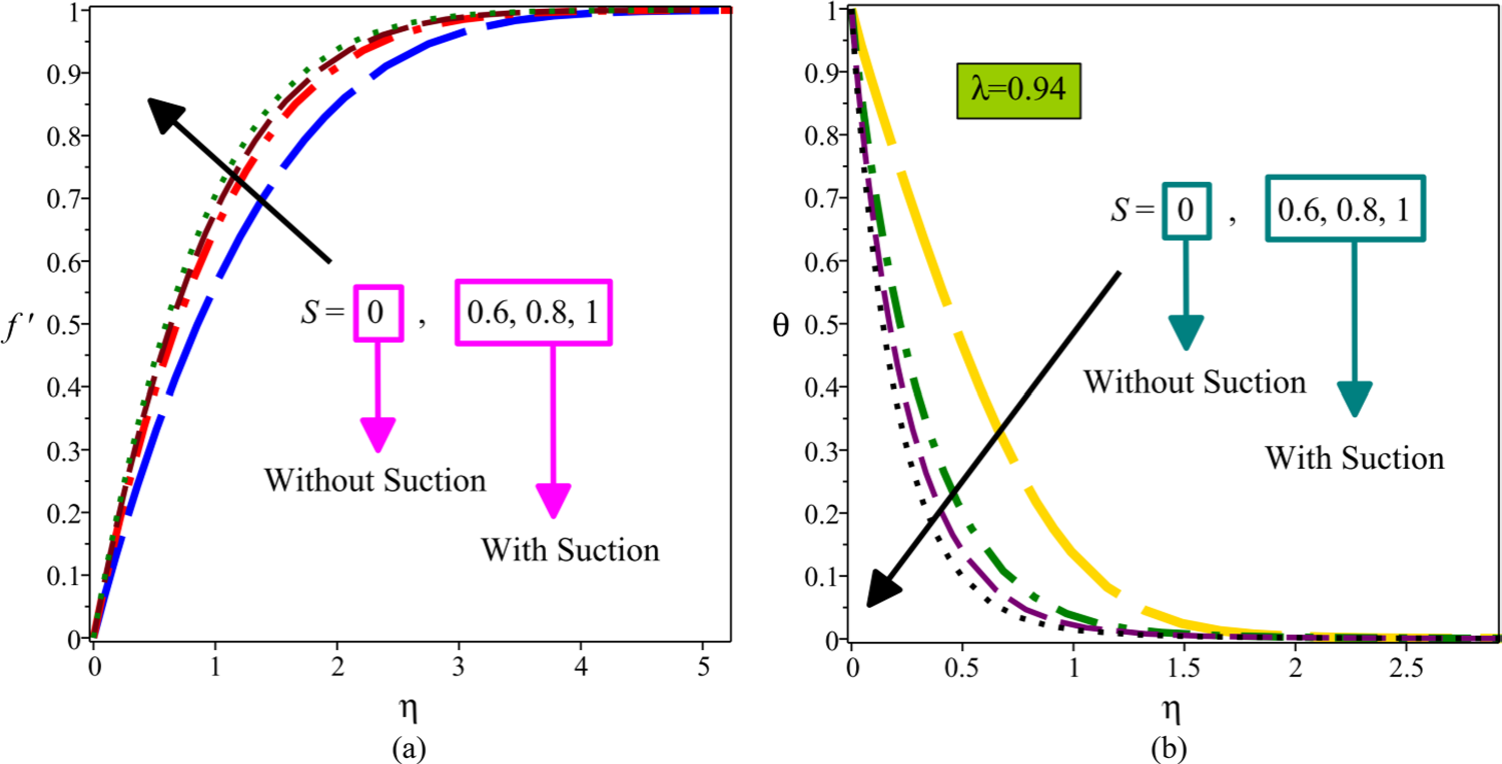
Velocity profiles (**a**) and temperature profiles (**b**) for various values of $S$.

Figure 3a,b are sketched to show the velocity and temperature distributions against the suction parameter $S$. As a physical result, using suction, part of the fluid is removed from the boundary layer by forcing it to flow via the permeable plate. Therefore, an increase in $S$ decreases both velocity and thermal boundary layer thickness. Thus, on growing values of $S$, the velocity of fluid increases but temperature decreases.

**Figure 4:**
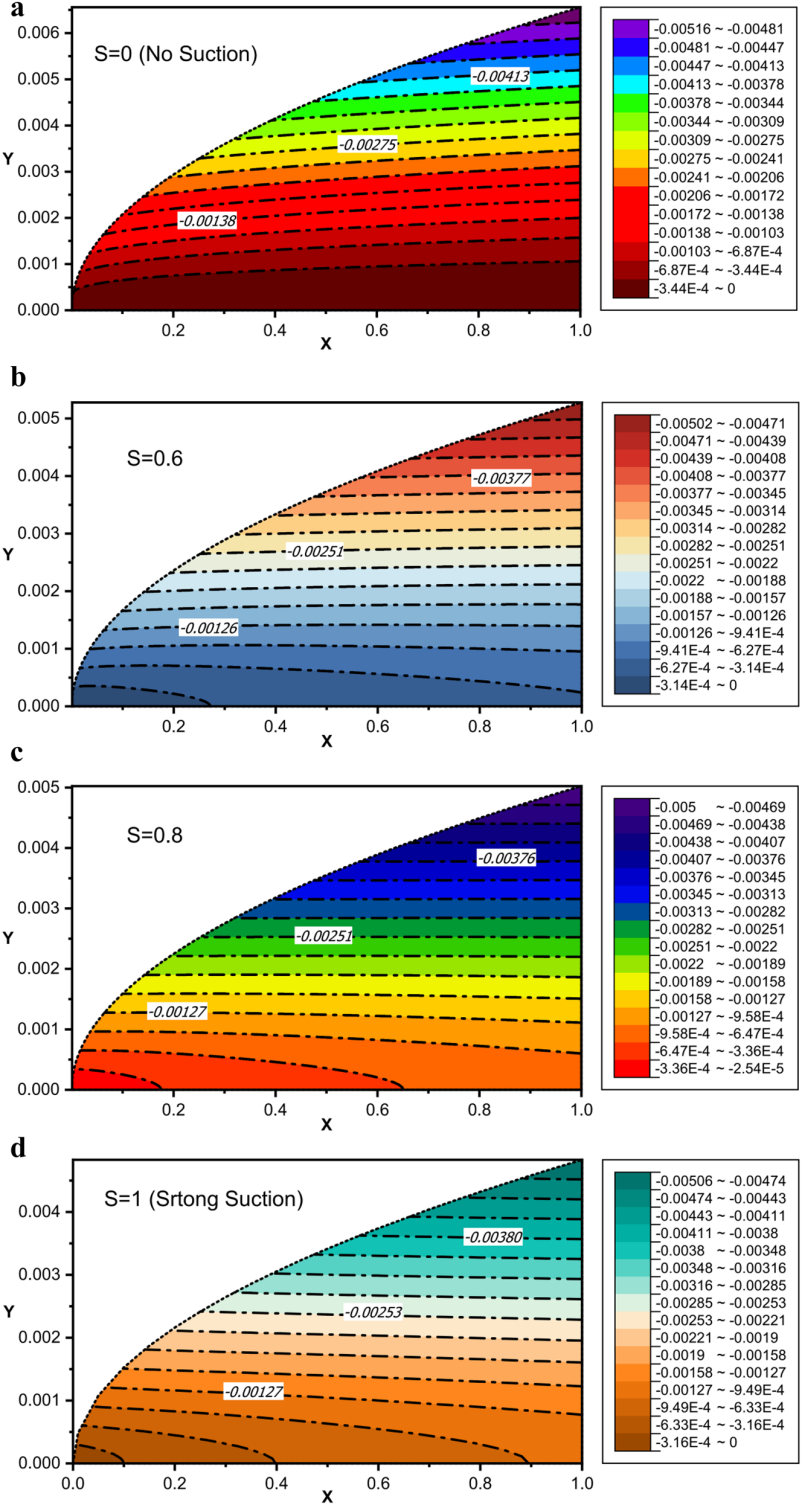
(**a**) Streamline contour without suction or injection ($S = 0$). (**b**) Streamline contour for $S = 0.6$. (**c**) Streamline contour for $S = 0.8$. (**d**) Streamline contour for strong suction ($S = 1$).

Figure 4a–d display streamline contours for the different suction parameter. For higher $S$, the suction intensity of the plate and consequently the vertical velocity are more strong when compared to smaller $S$. Therefore, as suction increases, the streamlines are drawn towards the plate, and boundary layer thickness decreases.

**Figure 5:**
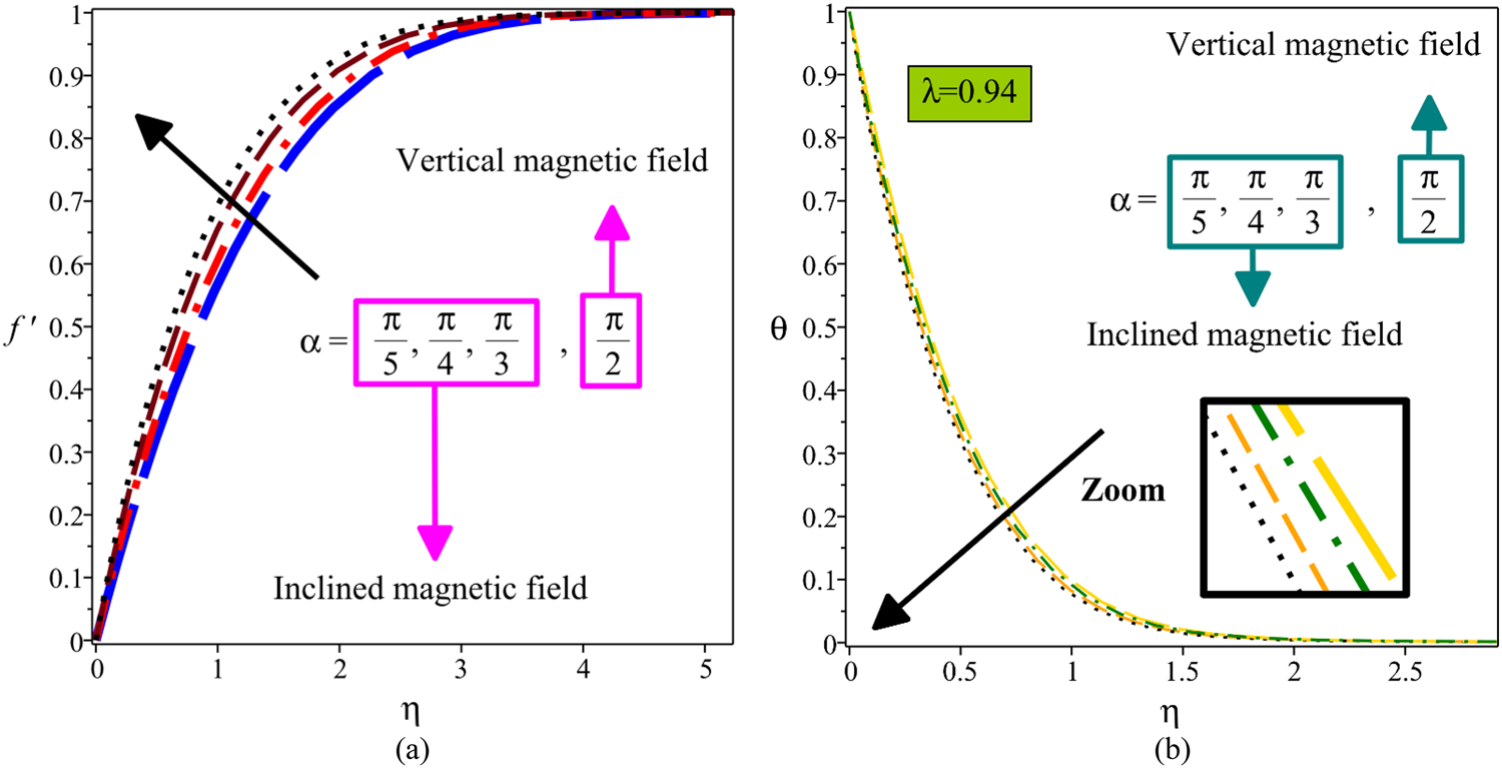
Velocity profile (**a**) and temperature distribution (**b**) for different values of $\alpha$.

The impact of the inclination angle of the magnetic field $\alpha$ on the velocity and temperature distributions is displayed in Fig. 5a,b, respectively. Here, when the magnetic field is perpendicular to the plate, the inclination angle of the magnetic field $\alpha$ is equal to ${\pi \mathord{\left/ {\vphantom {\pi 2}} \right. \kern-\nulldelimiterspace} 2}$. The interaction of the inclined magnetic field with the hybrid nanofluid flow produces the Lorentz force, which is a resistive force. As $\alpha$ rises from ${\pi \mathord{\left/ {\vphantom {\pi 5}} \right. \kern-\nulldelimiterspace} 5}$ to ${\pi \mathord{\left/ {\vphantom {\pi 2}} \right. \kern-\nulldelimiterspace} 2}$, the Lorentz force effect gradually increases. On the other hand, the favorable pressure gradient is amplified to overcome the Lorentz force under the Bernoulli principle. So, enhancement in $\alpha$ boosts velocity and decreases temperature. As a result, applying an external magnetic field with different inclination angles may be used as a mechanism to control the hydrodynamic and thermal behavior of hybrid nanofluid flow to achieve a desired performance.

**Figure 6:**
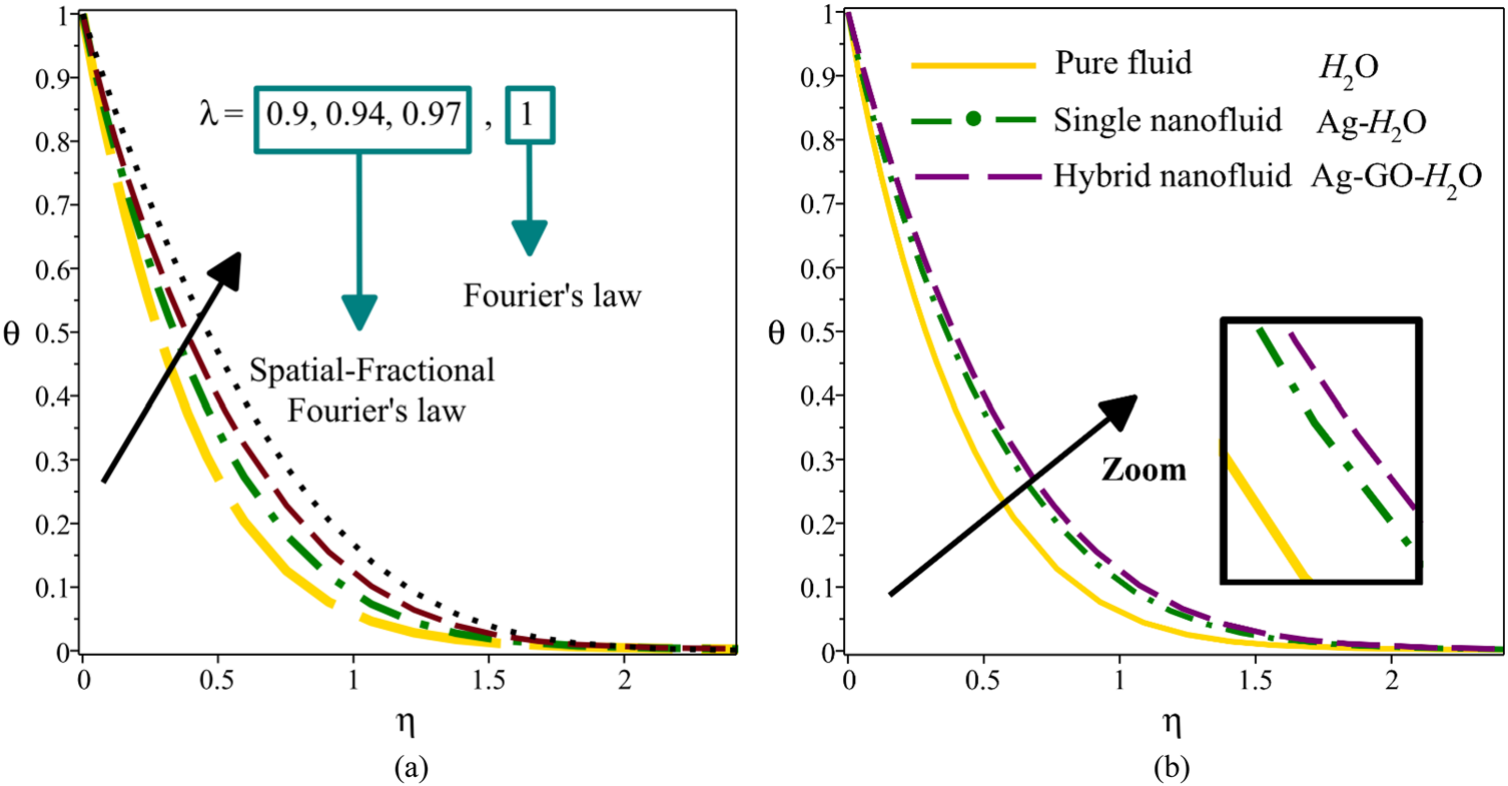
Temperature distribution for different values $\lambda$ (**a**) and for different working fluids (**b**).

Figure 6a compares the temperature profile for the spatial-fractional heat transfer model $(\lambda = 0.9,\,0.94,\,0.97)$ and its classical $(\lambda = 1)$. This figure show that an increase in the order of fractional derivatives increases the temperature of the hybrid nanofluid. On the other hand, the model of spatial-fractional has less temperature than its classical for describing the heat transfer process. It is to be noted that most research on fractional heat transfer has been focused on time-fractional derivatives, and less research has been done on space-fractional derivatives due to their complexity. Figure 6b displays the comparison between the temperature profile of the GO-Ag**-**H_2_O hybrid nanofluid ($\phi_{np1} = 0.06$, $\phi_{np2} = 0.06$) with the Ag**-**H_2_O single nanofluid ($\phi_{np1} = 0.12$, $\phi_{np2} = 0$) and H_2_O pure fluid ($\phi_{np1} = 0$, $\phi_{np2} = 0$). Based on the mentioned figure, the temperature of GO-Ag**-**H_2_O hybrid nanofluid is higher than both Ag**-**H_2_O single nanofluid and H_2_O pure fluid at all $\eta$ values.

**Figure 7:**
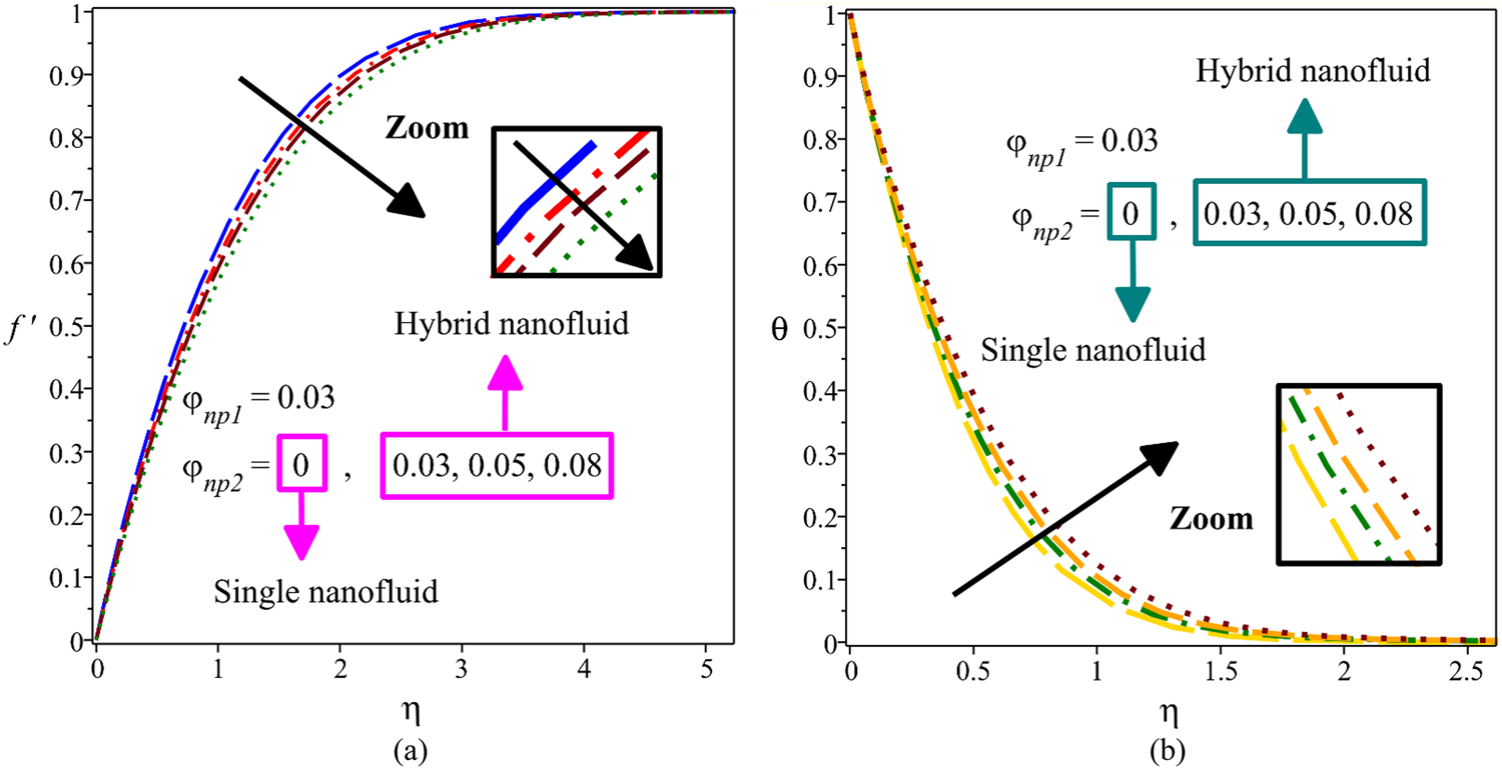
Velocity profile (**a**) and temperature distribution (**b**) for different values of $\phi_{np2}$.

Figure 7a,b illustrate the influence of variation the volume fraction of nanoparticles $\phi_{np2}$ on velocity and temperature distributions. Here, for GO-Ag**-**H_2_O hybrid nanofluid volume fraction of Ag is kept constant (i.e. $\phi_{np1} = 0.03$), and in the case of Ag**-**H_2_O single nanofluid, the volume fraction of GO is considered zero (i.e. $\phi_{np2} = 0$). Physically, when the graphene oxide nanoparticles are added to the silver-water nanofluid, the compound's thermal conductivity and kinematic viscosity increase. In fact, since the thermal conductivity of graphene nanoparticles is approximately eleven times that of thermal conductivity nanoparticles of silver and eight thousand times that of water ($k_{Graphene \, oxide} \, = 5000$, $k_{Silver} \, = 429$ and $k_{Water} \, = 0.613$), the addition of graphene nanoparticles to silver-water nanofluid, greatly increases the thermal conductivity of the compound. Furthermore, as the thermal conductivity of the compound increases, the diffusion of heat into the fluid flow increases (according to the first term of Eq. 24). The increment of heat diffusion in the fluid flow means an increase in the thickness of the thermal boundary layer, which results in an increase in the temperature profile. Similarly, the increase in volume fraction of graphene oxide nanoparticles raises the kinematic viscosity of the compound, leading to a thicker boundary layer and a lower velocity.

**Figure 8:**
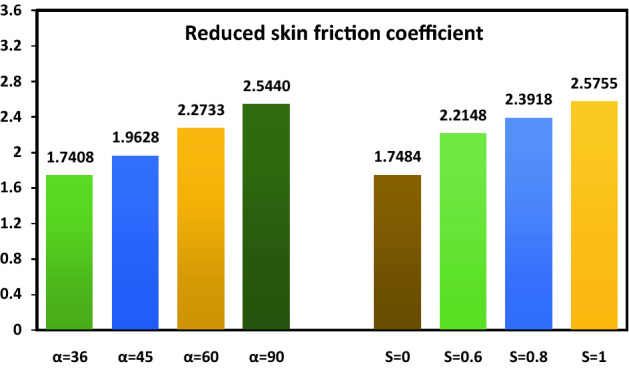
Values of the skin-friction coefficient for different $\alpha$ and $S$.

The physical quantity of interest, i.e., reduced skin friction coefficient, is calculated in Fig. 8 for several values of the inclination angle of the magnetic field $\alpha$ and suction parameter $S$. Physically, an increase in $\alpha$ and $S$ reduces the velocity boundary layer thickness, which will cause the enhancement of the velocity gradient on the plate. Thus, the skin friction coefficient decreases when $\alpha$ and $S$ are increased.

**Figure 9:**
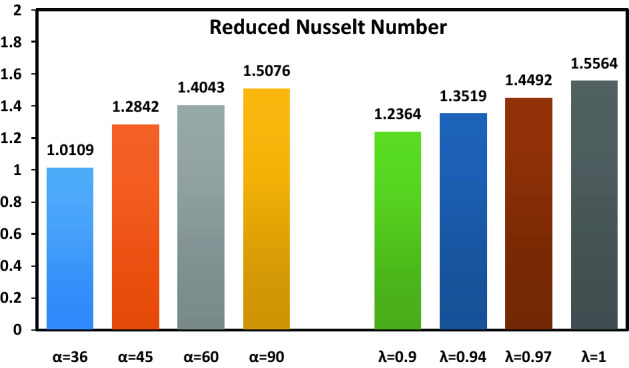
Values of the Nusselt number for different of $\alpha$ and $\lambda$.

Finally, Fig. 9 shows the variations in the magnitude of the Nusselt number for different values of the inclination angle of the magnetic field $\alpha$ and order of space-fractional derivatives $\lambda$. An increase in $\alpha$ leads to a thinner thermal boundary layer thickness resulting in a higher Nusselt number. Moreover, the value of the Nusselt number rises as the order of space-fractional derivatives increases. This physically means that the fractional model proposes a lower heat transfer rate than the classical model.

## Conclusions

In this paper, the space-fractional diffusion model is proposed for hybrid nanofluid heat transfer in the boundary layer flow along a permeable plate under an inclined magnetic field. The main points of this study are as follows:

- As an alternative to numerical methods, the optimized collocation method is successfully applied to solve the space-fractional boundary layer problems.
- The addition of GO nanoparticles to the Ag-water single nanofluid increases the temperature of the compound. This is due to the high thermal conductivity of GO.
- Increasing values of the order of space-fractional derivatives lead to a higher temperature and Nusselt number. So that, the heat transfer rate is augmented 26% by changing the order of space-fractional derivatives from $0.9$ to $1$.
- By increasing the suction parameter from $0$ (without suction) to $1$ (strong suction), the thickness boundary layer decreases, and the streamlines are drawn towards the porous plate.
- Changing the magnetic inclination angle from $36^{ \circ }$ to $90^{ \circ }$ (vertical magnetic field) causes an increase in velocity of the hybrid nanofluid flow.

Future research will focus on the study of influences of time–space fractional heat transfer and magnetic field within the boundary layer flow over a porous cylinder.

## Abbreviations

$q$: Parameter heat flux $\left( {{\text{W/m}}^{2} } \right)$
$$S$$: Suction/injection
$$Re$$: Reynolds number
$$B$$: Magnetic field $$(T)$$
$$k$$: Thermal conductivity $$\left( {{W \mathord{\left/ {\vphantom {W {m^{2 - \lambda } K}}} \right. \kern-\nulldelimiterspace} {m^{2 - \lambda } K}}} \right)$$
$$c_{p}$$: Heat capacity $$\left( {{\text{J/kg}}\,{\text{K}}} \right)$$
$\alpha$: Inclination angle of the magnetic field
$$Ha$$: Hartmann number
$$Pr$$: Prandtl number
$$\overline{X},\,\overline{Y}$$: Cartesian coordinates $${\text{(m)}}$$
$\overline{U},\overline{V}$: Velocity components in $\overline{X}$ and $\overline{Y}$ directions $({\text{m/s}}\,)$
$$c_{j}$$: Unfamiliar constants
$$R$$: Residual function
$$W_{i}$$: Weight functions
$C_{f}$: Skin-friction coefficient
$$Nu$$: Nusselt number
$G,Q,u$: Change of variable
$f\,^{\prime}$: Non-dimensional velocity

## Greek symbols

$\lambda$: Space-fractional parameter
$$\sigma$$: Electrically conductivity $$\left( {{1 \mathord{\left/ {\vphantom {1 {\Omega m}}} \right. \kern-\nulldelimiterspace} {\Omega m}}} \right)$$
$$\eta$$: Similarity variable
$$\psi$$: Stream function $$({\text{m}}^{2} {\text{/s}}\,)$$
$$\theta$$: Dimensionless temperature
$$\mu$$: Viscosity $$({{kg} \mathord{\left/ {\vphantom {{kg} {m\,s}}} \right. \kern-\nulldelimiterspace} {m\,s}}\,)$$
$\phi$: Nanoparticles volume fraction
$$\rho$$: Density $$({\text{kg/m}}^{3} )$$
$$\delta$$: Dirac function
$$\Gamma$$: Gamma function

## Subscripts

$$hnf$$: Hybrid nanofluid
$$nf$$: Nanofluid
$$f$$: Fluid
$$np$$: Nanoparticle
$$W$$: Wall
$$\infty$$: Ambient condition
